# Illinois State Dental Society

**Published:** 1885-12

**Authors:** 


### PROCEEDINGS.

### Illinois State Dental Society.

Meeting at Peoria, May, 1885.

### DENTAL PROTHESIS.

Dr. K. B. Davis, of Springfield, read a paper on the above subject in which he claimed that mere mechanics were not competent to operate at all in the mouth, not even to the extent of taking an impression.

After speaking of the uses of teeth, he said that artificial teeth might well serve for mastication, yet be defective in other respects, as in speech or in expression, which depends upon color, size, position, relative arrangement, etc. Temperament, including complexion, contour of face and general cast of features must all be considered in the construction of a set of artificial teeth that shall harmonize with the other features of the face, and constitute a successful work of art.

He favored the abandonment of plastic materials for bases, and blocks or sections of teeth; and in their places would use gold, platina and other metals for bases, and single plain teeth, with celluloid gums where artificial gums are required; prefers continuous porcelain gums on platina base for all full dentures. All healthy roots should be preserved and crowned, and bridge work employed to a limited extent.

DISCUSSION.

Opened by Dr. E. D. Swain, of Chicago. Webster defines the word prothesis as "The process of adding to the human body some artificial part in place of one that may be wanting, as a wooden leg," etc.

Regulating teeth and the taking of impressions are surgical processes, the construction of the appliances is mechanical.

Mechanical dentistry has greatly deteriorated of late years. Metal plates are now rarely constructed, the plastic bases having been largely substituted. The adaptation of metal plates can be made equal to that of the plastics if properly done, but that it is not now done except to a limited extent is evident. The use of plastic bases has become so common that very imperfect instruction in the working of metals is now given in most of the dental colleges. He believes, however, that this defect is gradually being remedied. All partial sets, in the schools and in practice, should be made of metal. For full dentures, continuous gum work is the king of methods, but its use is limited by reason of high cost and the superior skill requisite for its construction. Porcelain plates present too many difficulties, but they may be overcome in the future.

Cast plates are recommended for lower dentures.

Dr. J. R. Patrick, Belleville, spoke of taking impressions and declared it to be the most difficult of all the processes involved in the making of artificial teeth. The method he pursues is to first take an impression in wax ; on this he moulds a model plate of common red plate gutta-percha. The edge of this plate is stiffened by wire, when it is warmed around the edge and placed in the mouth ; directs patients to work the muscles of the mouth until a perfect adaptation of the edge of the plate is obtained to the muscles. The cup portion of the plate is then filled with plaster and then completed in the usual way. The impression of lower jaw is taken in a similar way. He regards air chambers as of no value after the first few days of wear, but likely to cause irritation, hence has ceased to make use of them.

Dr. G. V. Black said the subject of sore mouths found under rubber plates should be further investigated. This condition had been attributed to the coloring matter of red rubber plates, but it appeared under metal and porcelain plates also. A partial study of the causes of this disease has led to the discovery of leucocytes and micro-organisms under plates. In examining scrapings from plates and gums he had been surprised at the abundance of these organisms. If more abundant under rubber than under metal or porcelain plates, he was disposed to attribute the fact to the rougher surfaces of the rubber plates, which afforded greater facilities for the lodgment of foreign substances of a nature favorable to the growth of the organisms. Wandering cells—leucocytes—were seen constantly changing forms, micrococci aggregating around and among them. The wandering cells take up and digest the micrococci and thus aid in preventing the increase of the latter. Cleanliness is the chief preventive. The condition described differs from that of mercurial poisoning.

In reply to questions by Dr. Swain and others, Dr. Black said he did not regard the coloring matter injurious. The peculiar susceptibility which rendered one liable to mercurial poisoning did not exist in more than one case in ten.

The temperature of the tissues under a plate of non-conducting material varied from the normal less than one degree.

Dr. Swain had a case in which the worst case of salivation he ever saw was produced in thirty days after the insertion of a red rubber plate. The plate was left out and the salivation disappeared entirely, in two weeks The plate was replaced in the mouth when the salivation returned. Aluminum was then supstituted for the red rubber and no salivation followed the substitution.

Dr. C. W. Spalding, St. Louis. What is salivation? Salivation may be produced in five minutes, or, as in Dr. Swain's case, in thirty days. The sight of food may cause salivation or it may be produced by medicine in a very short time. The process is that of nervous excitation by reflex action. Pathological proceses do not differ from physiological processes, except in degree.

Dr. Black. Will you name a physiological process identical with ulceration?

Dr. Spalding. The cases are not parallel; ulceration is a death process and does not come within the range of physiology.

Dr. Noyes. The difference in ease of clensing between metal and rubber plates is one of smoothness. The rubber plate, being rough, is kept clean with great difficulty.

Dr. Sitherwood, of Bloomington, recommended aluminum as a suitable metal for plates, using rubber for attaching the teeth to the plates. It is cheap and good, and likely to succeed rubber as a base plate. The best he had obtained, came from France.

Dr. E. B. Call, of Peoria, said there was great difference in the quality of the samples of aluminum obtainable, but if a good quality of this metal was secured it would make a good plate.

Dr. W. W. Allport, Chicago, complimented the paper of Dr. Davis, but had expected something more on the artistic points in an artificial denture. Better arrangement of the teeth was needed so as to conceal the fact that the teeth were artificial. Size, color, and position are all essential, but color was a prime consideration. Dentists are deficient, as a rule, in a knowledge of the harmony of colors. They should study the art of coloring and shading and of harmonizing color for the best effect. The unpleasant aspect and faulty expression belonging to so many cases of artificial teeth are due to lack of knowledge on this point. A natural expression is the essential thing to aim at, and he hopes that study in this particular line will result in the production of a paper on this point next year.

Dr. T. W. Brophy, of Chicago. The area under the plate becomes engorged or congested, owing to an enlargement of the blood vessels, which lead to an over supply of blood. The view presented by Dr. Black is a later process.

The physiological process of absorption goes on for a long time after the teeth are removed, and for that reason plates should be renewed or altered every few years.

Dr. Taylor suggests a clinical demonstration illustrating the effect of teeth of varying color, size, position, etc., in the same mouth to be given next year.

Dr. I. P. Wilson, Iowa. I have seen similar results under gold plates that have been described as due to the coloring matter of rubber plates, and am of opinion that want of cleanliness is oftener the cause of the trouble than any specific poisoning proceeding from the material composing the base. If plates are not worn at night this abnormal condition of the tissues is not likely to occur.

Dr. Reed, of Chicago, described his method of procuring an accurate bite by substituting modeling compound for ordinary wax, when the discussion was closed and the subject passed.

Dr. G. Newkirk now read a paper, of which the following is a brief summary:

The peculiarly distinctive feature of nerve is in the relative proportions of its mass of nerve tissue.

The relative proportions and position of white and gray matter were considered; gray more distinctly cellular, white more fibrous. Gray more fragile, hence carefully protected. Various conditions of its protection: internal in cord, external in brain.

How may we understand something of the action of this inconceivable multitude of cells and fibres constituting the nervous mass?

Allowing the average size of the fibrilla to be 1-5000 of an inch, a nerve trunk containing the equivalent of a square inch upon section would have 25,000,000. The same number of telegraph wires lying side by side would fill four principle streets of Chicago fifty feet deep. Allowing forty wires to each person, it would require the service of every man, woman and child in the city to operate them.

But as we may understand the composition of the ocean by analysis of a single drop of water, so we may comprehend the principles of nervous action, generally by studying the character and action of a single series of cells and their connecting fibrillæ.

The writer then proceeded with the assistance of diagrams upon the board to show the relations of the central cell (in the cord, or brain, or ganglion) with the sensitive cell; by the sensitive nerve fibrillæ on the one hand, and the muscular or any contractile cell; by the motor nerve fibrillæ on the other; next the offices of the central cells of the cord as independent of the brain, then the economy of this arrangement.

The general functions of the gray matter of cerebellum and cerebrum was next considered. The wonderfully developed faculty which we call co-ordination of movement is undoubtedly resident in the cerebellum. How long a time it takes, and what a world of education of the central cells is involved in the difference between a toddling child and the bare-back rider or acrobat.

Even the ordinary movement of pedestrians on the street, as they come and go, presents the marvelous panorama of co-ordination.

But the crowning glory of man is yet beyond the cerebellum and medulla and striated body, in the gray cells of the cerebral convolutions.

Here dwells the king?

Other animals may be physically stronger, may possess equal or greater powers of muscular co-ordination, but in the one thing of cerebral gray substance, the organ of intellection and will, man stands alone, having dominion over all.

In conclusion, the author took the ground that there is no need of supposing any such thing as a "vital fluid," or "nervous fluid," or "electric fluid"—or anything which might be thought to travel along the nerves.

It is a matter of touch. The nerves, all the fibrillæ of the nervous mass, are simply millions of microscopic fingers with which the brain and cord are enabled to feel the things in which they possess an interest.

It is a "mode of motion." This point was illustrated by use of the telephone principle, etc. All these results are brought about by virtue of the fact that matter everywhere touches. The telegraph wire is merely an isolated line of atoms. By means of these the operators touch each other.

A man speaks, that is, he produces certain definite movements in the air toward a vibratory membrane, the membrane touches a wire, the wire another membrane, the membrane the air, again, the air the ear of man No. 2. So by means of atoms in contact the men touch each other in such manner as to convey and receive information, and this is the telephone.

So with nerves; the sensitive cell is merely the terminal cell of a series of cells, all in contact. The first touches the second, the second the third, and so on, till the central cell is reached.

DISCUSSION.

Discussion opened by Dr. Judd.

Grey nervous matter is found in the sensorium, the spinal cord, and other nervous centres. This structure is made up mostly of cells, and the cells are surrounded by a gelatinous envelope or stroma as an additional protection against injury. As the writer stated, the mass of gray matter is carefully protected, but nature also protects each individual cell. I doubt if animal cells, however carefully and finely wrought, can ever originate thought. Thought is not brought into existence by any physical means. It is more or less divine in its nature, and indicates the presence of Omnipotent omniscience.

Dr. Spalding. It is difficult to discuss the subject under consideration without stepping outside the limits of the physical and into the domain of the psychical. The most eminent minds in the scientific world have long endeavored to account for mental phenomena upon mere physical grounds. In my opinion this attempt has always been a failure, and I avail myself of the opportunity to antagonize this view. There are no chemical or other physical processes taking place in the brain that can possibly originate a thought or any other mental process. There is no molecular change however subtle that can produce a mental result. Thoughts, and all mental activities, come to us from the mental world—the world of spirits; and not from the world of nature. The fact is, the spiritual world is, at all times, just as near to our spirits as the physical world is to our bodies.

The paper refers to the differences between the young of animals and infants. This difference is so great that a comparison can scarcely be drawn. Animals come into the world capable, in great degree, of taking care of themselves; they are born with certain knowledge. They can stand and walk and search for their food at the mother's udder almost as soon as they are brought forth. Man, on the other hand, is utterly helpless at birth, his mind is blank, he is born without knowledge and is therefore capable of acquiring all knowledge.

The question was here raised whether the discussion was not traveling beyond the subject matter contained in the paper.

Dr. Judd said the question whether grey nervous matter can originate thought is a legitimate topic for this society to discuss. It is difficult to do this however without trespassing upon the domain of the spiritual. The spiritual and the physical come in contact through the grey matter of the sensorum.

Dr. Taylor. Will Dr. Newkirk explain how we get morbid reflex action?

Dr. Newkirk. This occurs through the association of the central cells with those of the peripheries, there is a faulty registration.

Dr. Morrison. It is a crossing of the wires.

Dr. Newkirk. Yes! The same as sometimes occurs when the kiss a fellow sends on a wire goes to the wrong party.

Dr. Black. With regard to this misplaced reference of pain or reflex pain as it is called, did you ever know such a thing as reflex pain when the sense of touch was implicated in the impressions? Touch is the great localizer in the nervous system; it has no other. When touch is involved the central cells have no record of an impression. There is no pain in the liver or the cornea, etc. The tooth pulp has no sense of touch and hence is incapable of localizing pain. The sense of touch is requisite for the localization of pain in any part of the body. Pain in the knee in hip disease, is an illustration. The pulp has the power of transmitting sensations of pain only, and is utterly incapable of distinguishing between heat and cold, as I have frequently demonstrated. The peridental membrane has the sense of touch.

Darwin demonstrated reflex action to be not within the control of the will.

Dr. Spalding. Reflex action often occurs independent of the will or even of the brain. There are other nervous centres besides the brain, and reflex action may be referred to subsidiary nervous centres. The eye may wink as an involuntary act before the will becomes cognizant of danger to that organ. There is a common idea that there exists somewhere in the body a life centre —a place, or an organ, where life especially resides. I apprehend that life pervades the entire body, that every organ receives life of a degree corresponding to its importance. If life did not pervade the whole body, how could the body in all its parts be under control?

Subject passed.

Dr. Geo. H. Cushing read a paper on Operative Dentistry in which he pointed out some of the causes of the failure of fillings and urged the importance of great thoroughness in all operation on pulpless teeth. He said formerly, filling cavities of decay, extracting teeth, and removing salivary calculus comprised the full range of the duties of dental practice. But now, sympathetic relations are recognized as existing between the teeth and other organs of the body, and diseases of the teeth affect other organs to such an extent that a good knowledge of medicine is required, that the dentist may be able to successfully meet and relieve such conditions.

Formerly, creasote and opium constituted the bulk of the dentists' materia-medica, but at this time thirty or more medicines are in constant use and every dentist is expected to be competent to properly dispense them. Fractures of the jaws and the removal of tumors are also included in dental practice.

Diseases must be studied and a good knowledge of materia-medica and therapeutics is essential to an intelligent practice. A fair knowledge of general, and an acute knowledge of special anatomy and physiology is required, and an ability to apply the principles of general surgery is also essential. A first requisite is a high degree of mechanical skill, coupled with delicate manipulative ability; and to acquire these a long tuition and close observation are necessary. As to the causes of the failure of fillings there are several: one frequent cause lies in the defective preparation of cavities. Often they are not sufficiently enlarged especially at their margins. This is most liable to be the case with cavities so located that their thorough examination is difficult. There is a zone around cavities of decay—an infected area, that is liable to break down unless it is removed. A lens greatly assists in determining the extent of this area, and its use is always advisable in determining the condition of a cavity previous to the introduction of any filling. The fillings themselves are sometimes defective from overlapping, especially at weak points. These weak walls should be cut away, and particular attention should be given to the preparation of the cervical margins of all proximal cavities.

There exists also a strong tendency towards over-malleting. Less force is required to insure compactness than is commonly supposed. Soft gold and hand pressure should be employed until the margins are covered with sufficient gold to afford ample protection against the impact of the mallet. Crowns should be so contoured as to leave open spaces at the gum lines, with a view to promote cleanliness.

Dentists are liable to allow themselves to be hurried, and thus attempt to do in one hour that which would require two for its proper execution.

These ideas are not new, but they must be reiterated—must be repeated again and again, to ensure proper observance.

Pulpless teeth require special care. There is a great ignorance among medical men concerning pulpless teeth, and many of their published views are absurd, and are quite contrary to the experience of intelligent dentists.

The fault among dentists, however, is not a lack of knowledge relative to the proper treatment of such teeth; at least the teachings on the subject have been ample and in the main correct; the chief difficulty lies in a lack of ability to successfully apply the theories taught. There is also a lack of thoroughness in the work. Treatment becomes irksome, and consequently root filling is often permature. Operators who do this permature work are likely to also make imperfect work when the root canals present unusual difficulties. I insist that the greatest certainty must be had in every step in this process, and exceeding thoroughness must prevail in the entire operation.

DISCUSSION.

Dr. Taggart. Thoroughness is a prime essential in all operations upon the teeth. The dentist should make no imperfect work for lack of time, but time enough should be taken, and a fee charged corresponding with the time consumed.

Soft gold (so called) spreads more rapidly in the cavity than cohesive gold, but I doubt whether it is any better than cohesive for any purpose.

Physicians are not competent to judge of either the value or the savability of pulpless teeth. Dentists are quite ready enough to condemn teeth when they are past saving, and a better knowledge of therapeutics would doubtless enable them to save many that are now lost.

For use in root canals I prefer broaches of about the temper of piano wire. Barbs upon them so increase their diameter that they can be used only in the largest canals. Smooth broaches are necessarily used in the small canals; they should be draw-filed to prevent cross grooves. The canals must be throughly cleansed, for there are no medicines that will compensate for imperfect cleansing. Great care should be taken to avoid the projection of any filling material beyond the apex of the root. It is claimed that such foreign materials will become encysted. It is true that leaden bullets are sometimes encysted, but it is also true that many who are wounded with bullets die, because the bullets do not become encysted.

Dr. W. O. Kulp, Davenport, Iowa. Inserts piano wire in a socket which having been heated by being rotated in the lath, shrinks upon the wire and thus holds it firmly. The wire can be then reduced at will. Uses no other broaches. In the preparation of cavities for gold fillings, the margins must not be left sharp, but should be beveled to prevent leakage. The complaint of disturbance from pulpless teeth that has appeared in some of the medical journals is largely due to the practice of capping pulps.

Dr. H. J. McKellops, St. Louis, says that No. 10 Swiss broaches are so delicate that no one not expert can make them. We cannot afford to spend the time required to make our own broaches when they can be bought so cheaply. I use barbed broaches in clearing the root canals. I think Dr. F. M. Badger, of New Orleans, was the first to use broaches for this pupose. Donaldson's bristles are good, but not as convenient to use as the Swiss broaches. These broaches are tempered in an iron box, unslacked lime being used to fill between, around, and over the broaches. The box and contents are then heated until the box is read hot, when the mass is allowed to cool.

I regard Jack's matrices as a valuable aid in doing first-class work in those cases where such an implement is required; and waxed tape I value as an efficient and convenient agent for separating the teeth.

Dr. G. Newkirk demonstrates his method of wrapping broaches with cotton. The fibres of cotton should be held longitudinally with the instrument, and the wrappings should be finished at the end of the broach.

Dr. W. N. Morrison, St Louis, is opposed to reaming or in any way enlarging root canals. The instrument employed should be made to fit the canals, and not the reverse, and should be a little smaller than the canal into which it is to be passed. The five sided broaches as they come to us from the manufacturer are rotated in the canals with some difficulty, by reason of the sharpness of the corners. These corners are designed to cut, and as we do not use them for this purpose the corners should be slightly rounded or dulled to favor easy rotation.

Dr. L. C. Ingersoll, Keokuk, Iowa. We bestow too much attention upon the methods of operating on pulpless teeth, and fail to properly consider the treatment of the peri-dental membrane belonging to these teeth. If this membrane is preserved in a healthy state or restored to health after being diseased, the tooth may be easily preserved. In all cases a dead pulp implies disease of this membrance, and this condition must not be disregarded, if we would preserve the tooth as a useful organ. The foramen at the apex must be closed, or as nearly so as is practicable, unless nature closes it with cementum, as is sometimes done. You cannot expect success unless this passage is sealed. I have found cases where the broach could be readily passed through the foramen, and passed it many times, and yet at the end of three months I found nature had closed this opening, and the same broach which formerly passed with ease would pass no longer.

Dr. G. V. Black. The paper states that we do not cut away sufficiently to remove all the affected area in preparing cavities. This is especially true of the posterior surfaces of second molars. On the proximal surfaces of bicuspids, also, we often fail to cut away laterally as much as we should do. So much of the enamel as is at all whitened, be it ever so slight, should be removed. The dentine may be unaffected, but the enamel is injured where this appearance is present. If these teeth are examined after extraction, you will find that a delicate shaving when magnified will show the injury distinctly, and will be found to be infested with micro-organisms. Such cavities should, therefore, be enlarged at the base until all the injured enamel has been removed. This whitened aspect of the enamel is produced by a pathological condition of the gum tissue or by micro-organisms.

Dr. Cushing asks if a zone of enamel beyond what can be discovered is not affected.

Dr. Black thinks that injury to enamel does not extend beyond what a magnifying glass discloses.

Dr. Brophy asks how much of this injury is due to the action of lactic acid

Dr. Black answers: "You cannot get this condition except through the agency of micro-organisms."

Dr. J. N. Crouse. The practice persued in any given case should be adapted to the kind of teeth to be treated, and to the care the teeth and your operations are likely to receive in the future. Bell-crowned teeth must be wedged apart, for if cut away, they will probably come again into contact, and if cut much are seriously impaired for usefulness in mastication. Permanent separations between teeth of this shape should not be made. As to wedging, patients can be educated to do much of this work themselves. Don't advise large and expensive operations in filling, unless there is a reasonable hope that the work will be taken care of afterwards.

Children can, by a proper course of training, I mean training by the dentist, be brought to the point of becoming interested in the condition of their teeth, and of keeping them clean. This requires repeated instruction and actual demonstrations by the dentist. Show children how to cleanse their teeth and require them to do it in your presence. Manifest an interest in their teeth yourselves, if you expect to arouse a corresponding interest on their part.

The preventive treatment of teeth, that is, to prevent decay or re-decay, is a most difficult thing to decide upon. Sometimes permanent separations are best, and sometimes they are prohibited by the forms of the teeth, or by conditions arising from other causes.

In the treatment of exposed pulps I have made no change in practice. I still continue to conserve pulps by capping.

The piano-wire broach has a stiffness that is not possessed by the Swiss broach after it has been annealed, and for that reason it can be passed into canals, into which the common broach will not readily enter.

As to the kind of gold foil best to use, I think it advantageous to my patient and to myself to use non-cohesive gold for a large portion of a filling above the average in size. Retaining pits are not necessary in the use of non-cohesive gold, grooves being substituted; and, as retaining pits are, as a rule, objectionable, that is an additional reason for the preference for this form of gold.

A good and useful operation may be made by the use of tin for half or more of a large filling, and finish with gold to give a durable and elegant surface.

Dr. Kulp, when asked if he thought exposed pulps were saved alive by capping, answered, "Not many."

Dr. E. Noyes. Gold cannot be beaten in the cavity as it is by the gold beater. Gold can be wedged into cavities, and into grooves and other irregularities in their walls, but I don't think the molecules of gold can be made to change their relation to each other; that is, gold is not spread by the mechanical force applied to it in filling.

Dr. W. W. Allport. The spreading of gold depends on the force applied and the form of the instrument employed. If flat surfaces are employed, the gold is not spread, but if rounded surfaces are forcibly applied, it is. Non-cohesive gold is more easily adapted to inequalities of surface than is cohesive, and this adaption constitutes the chief difference in the two kinds. I am sorry to hear Dr. Black advocate cutting away as much as he does. It does not seem to me to be either necessary or advisable.

When the two metals, gold and tin, are used in the same filling, I prefer that they be combined, that is the two foils mixed or twisted together and inserted in this relation to each other. There seems to follow a peculiar union of the metals, something like an amalgamation of them, for when such fillings are removed after having been worn a number of years, we find this union such that the two metals cannot be separated mechanically.

Dr. A. W. Harlan. A principal cause of failure in fillings is a lack of a systematic method of practice. If you allow yourself to be interrupted by callers when your time has been previously engaged to some one else, you will become hurried, and your work is liable to show it. In my practice I do not allow this. I reserve certain hours for callers, and unless they call at the proper time, they are not seen by me, but must transact their business with an assistant, or call another day. Medical men vainly attempt to define the duties of the dentist. Many of them cannot distinguish a pulpless from a living tooth, nor a deciduous molar from a permanent one.

Dr. W. B. Ames. I began my practice with the use of cohesive gold, and when I examined my failures for the purpose of discovering the cause, I found the failures mostly happened in cavities that were difficult of access, or in portions of cavities not easily accessible. In such situations I now use non-cohesive gold with improved success. Gold spreads in the cavities by one layer sliding upon another, and not otherwise.

Dr. G. D. Sitherwood. Physicians have not studied the conditions present in pulpless teeth because the treatment of such teeth is out of the line of the general practice of medicine.

For fillings that are subject to much friction in mastication I use the so-called platinized gold, and find it much more durable than gold alone.

Dr. L. Ottofy. I don't think gold spreads; that is, the molecules do not move upon each other; but masses of non-cohesive gold flatten out, and thus are spread under the mallet.

Dr. Black. Water is spread by being pressed upon; the plumber spreads the lead used in securing joints; why cannot the dentist spread gold upon the same principle?

Dr. C. W. Spalding, St. Louis. Gold spreads under the force of the blows of a mallet, just as it and other metals spread in the act of forging. I mean that gold may be forged in the cavity of a tooth, provided the proper force is applied in the right direction. After packing a filling, if you are doubtful about the margin being tight at all points, just go over the surface of the filling with a suitably shaped instrument, and you will find the gold can be readily driven against the wall-margin without injury to the tooth. This is not an opinion; it is a matter of repeated demonstration, and one that I constantly practice. The point of the instrument should of course be placed near the edge of the plug, and the proper shape of this point will suggest itself to every intelligent mind.

Dr. H. J. McKellops does not agree with the last speaker. He does not think cohesive gold can be spread after it has been properly consolidated in the cavity.

Memorial resolutions were then passed on the death of Dr. H. N. Lewis, of Quincy, Dr. D. B. Baker and Dr. S. H. Verbeck.

Dr. Sturges, of Quincy, spoke of the long and conscientious devotion of Dr. Lewis to his professional duties, and his earnest labors for the advancement of dental science.

(TO BE CONTINUED.)

### Proceedings and Discussions of the Ohio State Dental Society, Re-organized at Chillicothe, Ohio, October 28th, 29th and 30th, 1885.

